# Bizarre Parosteal Osteochondromatous Proliferation (Nora’s Lesion) in the Hand: A Case Report and a Review of the Literature

**DOI:** 10.7759/cureus.50439

**Published:** 2023-12-13

**Authors:** Sultan N Alaqil, Rakan H Alelyani, Othillah M Moazin, Hussain Alobaidi, Emad A Alfadhel, Dana Al-Kadi, Haneen H Baitalmal, Tanveer A Bhat, Mohammed Y Mirza

**Affiliations:** 1 Department of Plastic and Reconstructive Surgery, King Saud Medical City, Riyadh, SAU; 2 Department of Pathology and Clinical Laboratory Administration, King Saud Medical City, Riyadh, SAU

**Keywords:** bone tumor, painless mass, metacarpal bones, hand, bizarre parosteal osteochondromatous proliferation, nora’s lesion

## Abstract

A Nora's lesion, a rare and typically non-cancerous growth originating from the parosteal osteochondromatous tissue, primarily manifests in the hands and feet. Despite its benign nature, diagnosing Nora's lesions is complex due to their tendency to exhibit aggressive features in imaging scans and the ambiguity encountered in histological examinations. This rarity is evidenced by the limited number of reported cases in medical literature since its initial discovery. Detailing a distinctive instance, we document a specific case of a Nora's lesion situated on the dorsum of the left hand, specifically above the shafts of the fourth and fifth metacarpal bones. Through a meticulous histopathological analysis, the diagnosis was confirmed, aligning precisely with imaging features. To address the lesion conclusively, a comprehensive surgical excision of the mass was performed. This particular case not only adds to the scant body of documented instances but also underscores the significance of accurate diagnosis and management. Understanding and documenting such cases are crucial in refining diagnostic approaches and optimizing treatment strategies for Nora's lesions, emphasizing the ongoing need for further research in this domain.

## Introduction

Bizarre parosteal osteochondromatous proliferation (BPOP), commonly referred to as a Nora's lesion, was first delineated in the medical literature by Nora et al. in 1983 [1]. This peculiar benign growth stems from the osteochondromatous tissue and predominantly manifests within the extremities, notably in the hands and feet [2]. Despite their benign nature, Nora's lesions exhibit an intriguingly aggressive local growth pattern [3,4], often posing diagnostic challenges due to their rarity and atypical presentation.

The scarcity of documented cases confines our understanding of Nora's lesions to sporadic reports or small case series, impeding the establishment of standardized treatment protocols. Notably, post-excision recurrence is a prevailing concern, contributing to a notably high recurrence rate [4,5]. This recurrent nature underscores the complexity of managing Nora's lesions and underscores the necessity for comprehensive, long-term follow-up care.

The deceptive nature of its presentation may lead to misdiagnosis or delayed identification, potentially resulting in inappropriate initial management. Such delays in diagnosis could necessitate more aggressive surgical interventions, such as ray amputation, impacting not only the physical appearance but also profoundly influencing the functionality of the affected limb [6].

In this report, we present a compelling case involving a distinct mass located on the dorsum of the left hand. Subsequent histopathological evaluation confirmed the presence of a Nora's lesion.

## Case presentation

**Figure 1:**
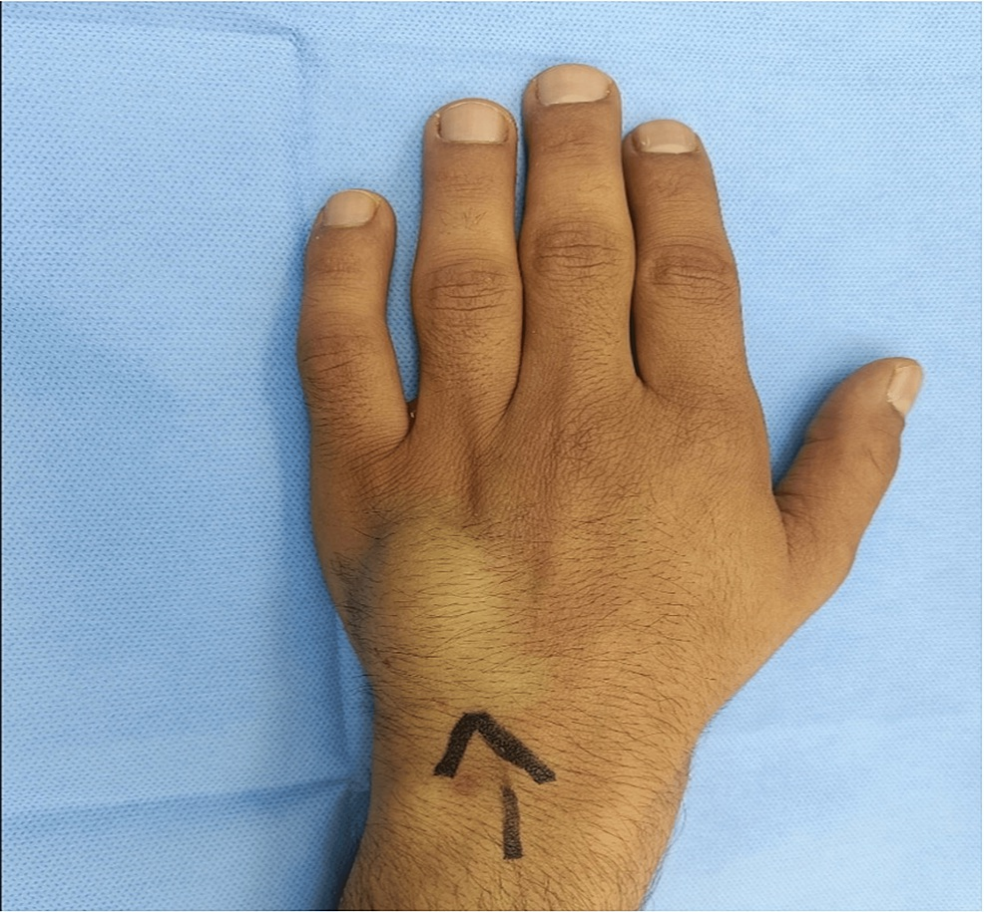
Image showing swelling on the dorsum of the left hand

A 35-year-old male patient attended our plastic surgery outpatient department (OPD) with a history of painless progressively growing swelling over the back of his left hand for the last two years ( Figure 1). The swelling was not causing any problem to the patient in doing his routine activities of daily life (ADL); however, the progressive growth of the swelling and the resultant cosmetic disfigurement concerned the patient. There was no history of any other swelling in the body nor there was any such family history. Moreover, there was no known history of trauma to the left hand. On general physical examination, the patient was stable and apparently in a normal state of health. Local examination showed a visible peach size swelling on the dorsum of the left hand lying over the shafts of the fourth and fifth metacarpal bones. The overlying skin was normal on inspection without any signs of inflammation. On palpation, the temperature of the swelling was comparable to the surrounding skin, and it was non-tender, bony hard to feel with discrete margins, and measuring approximately 3 cm × 2.5 cm in size. The swelling was mobile more in the vertical plane than in the mediolateral plane. The overlying skin was healthy and freely mobile. The distal neurovascular status and the range of motion (RoM) of the hand were within the normal range.

**Figure 2:**
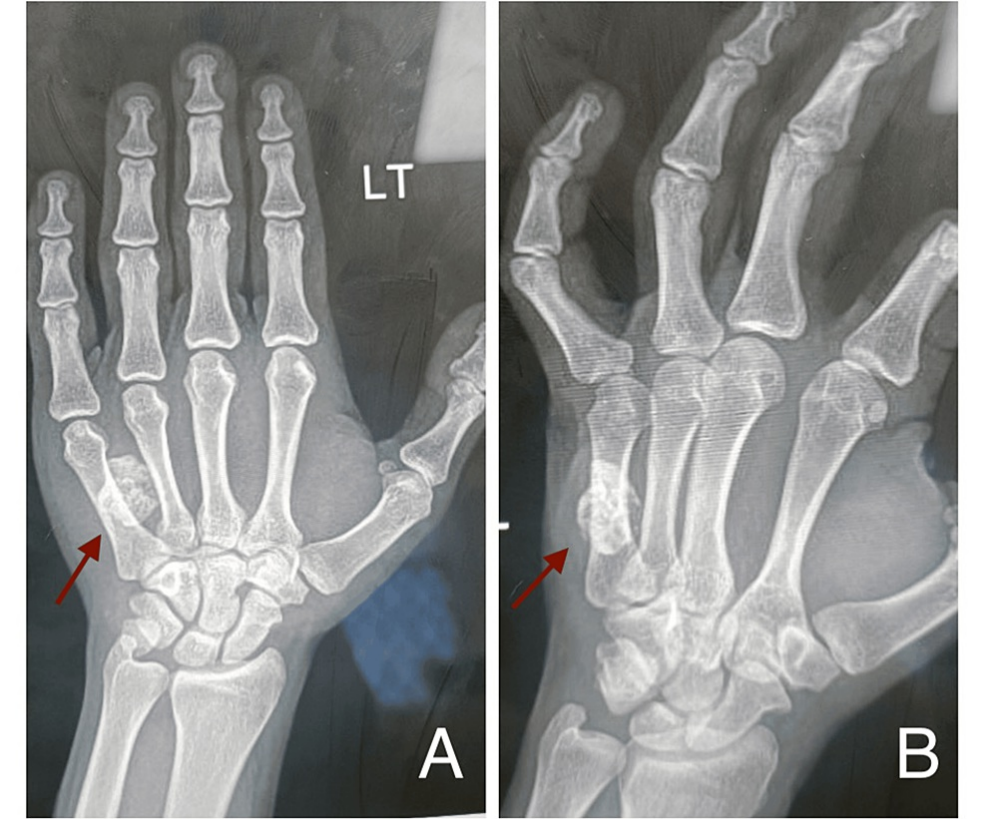
Image showing the anterior-posterior (Figure A) and oblique (Figure B) X-ray views of the left hand with the calcified lesion over the shaft of the fifth metacarpal bone extending to the fourth inter-metacarpal space.

X-rays of the hand showed a swelling with dense calcifications just lying over the shaft of the fifth metacarpal bone and extending to the fourth intermetacarpal space radially (Figure 2). The calcifications were not uniform throughout the swelling. The metacarpal bones were normal in size and shape and apparently normal in outline without any erosions or irregularities. A provisional diagnosis of osteochondroma was made. Baseline investigations were done and found to be in the normal range with a hemoglobin of 13 gm/dl. The treatment plan was explained to the patient, an informed consent was documented, and the patient was booked for surgery as a daycare case. 

**Figure 3:**
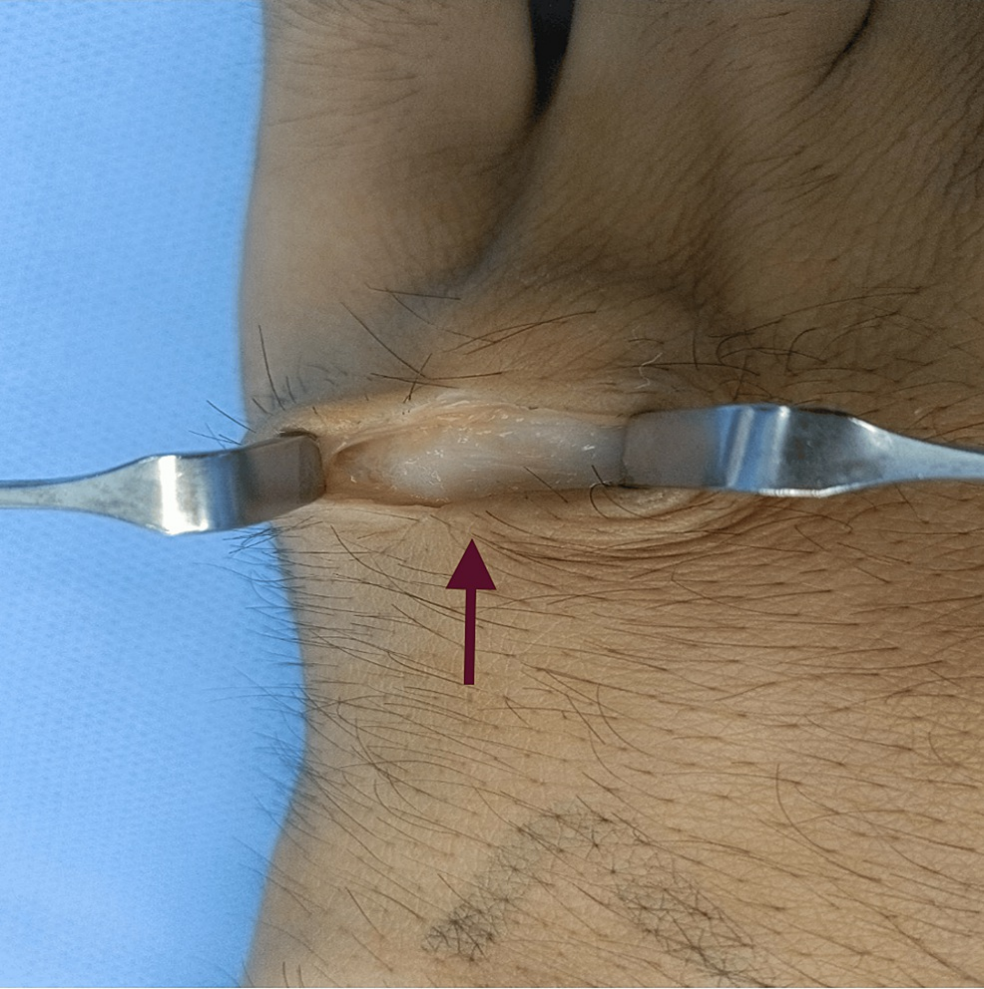
Intraoperative picture showing a pearly white swelling in the subfascial plane

**Figure 4:**
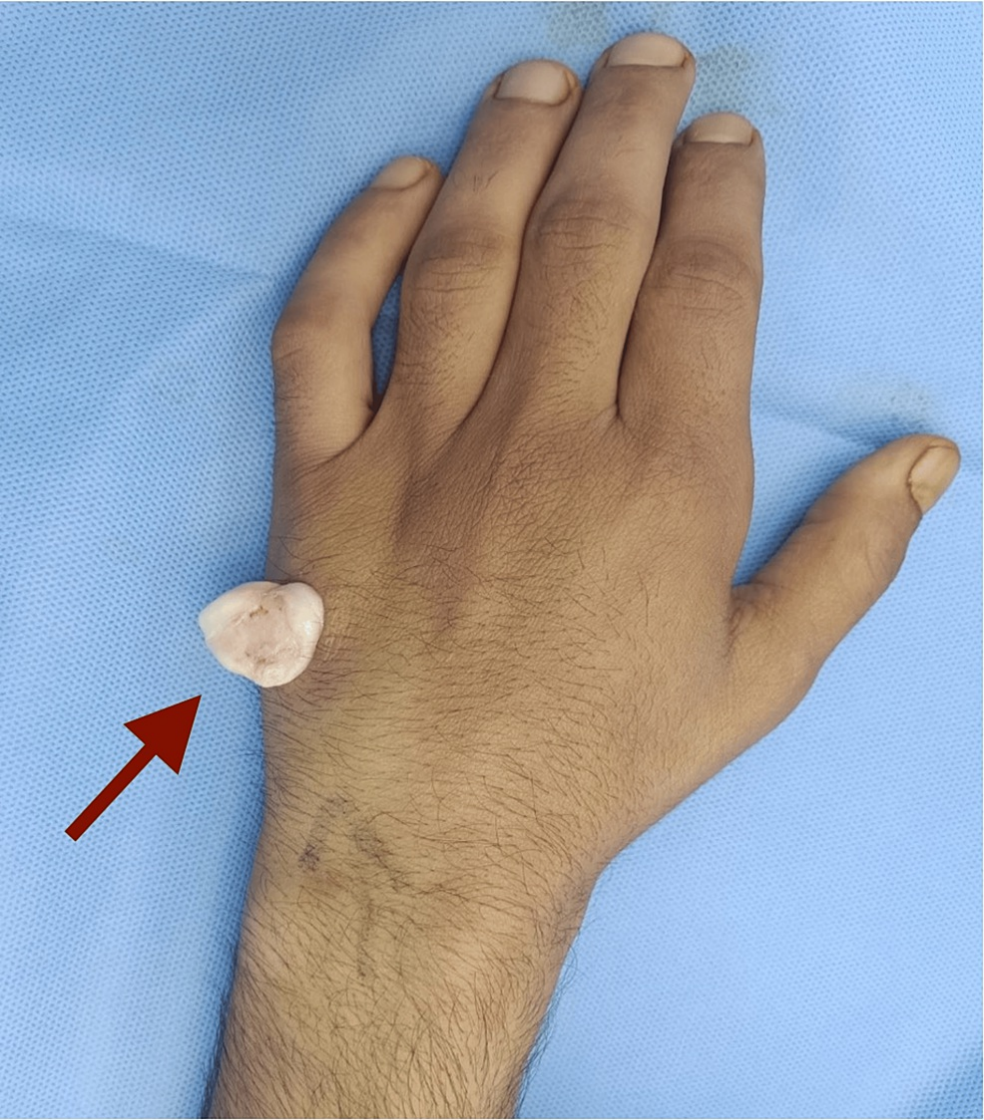
Intraoperative picture showing the dissected-out swelling

**Figure 5:**
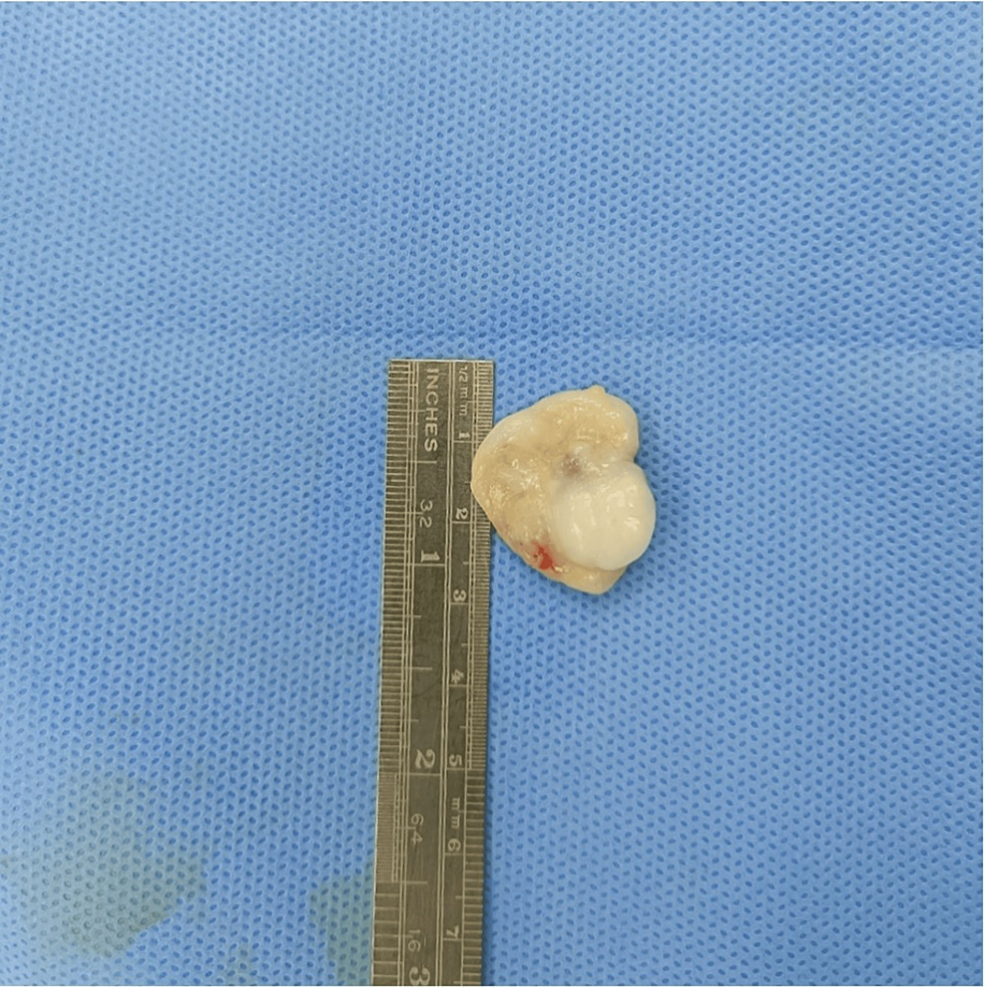
Picture showing the excised swelling with apparently two different tissue types

**Figure 6:**
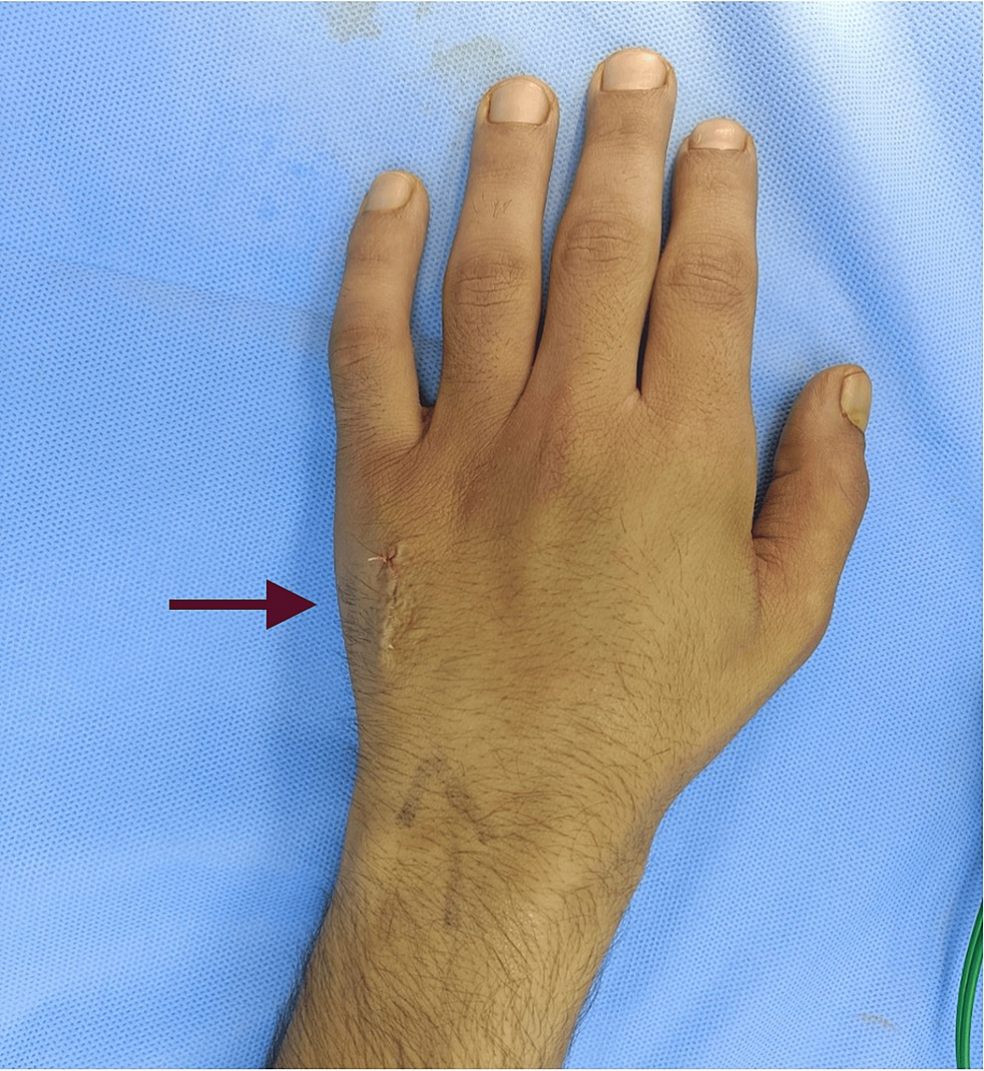
Intraoperative picture showing the wound closure after the excision of the swelling

**Figure 7:**
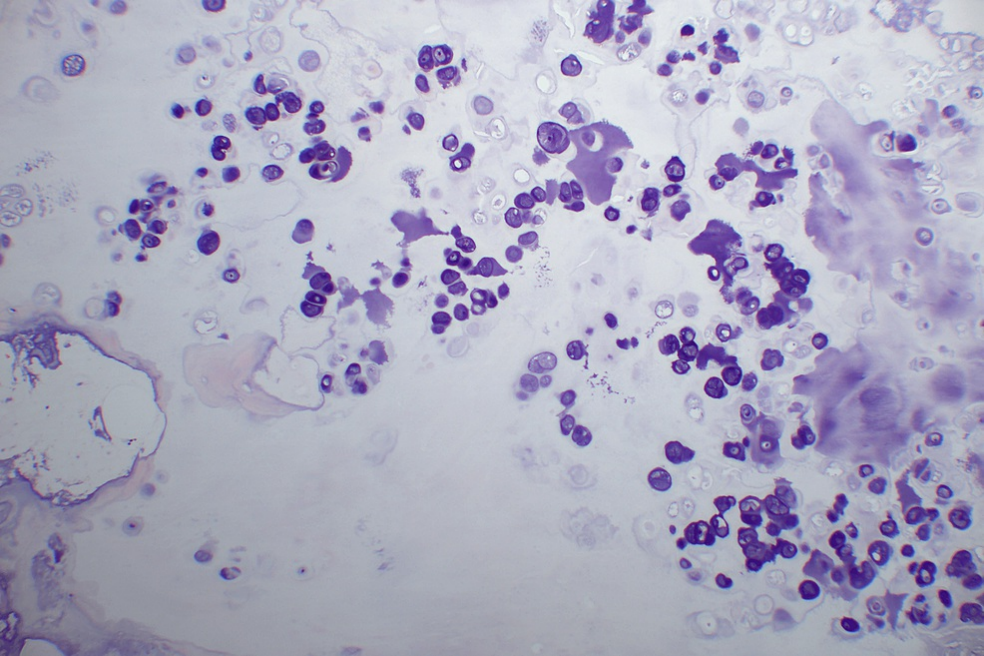
Osteocartlignous proliferation with classic basophilic areas of a “blue bone” formation

**Figure 8:**
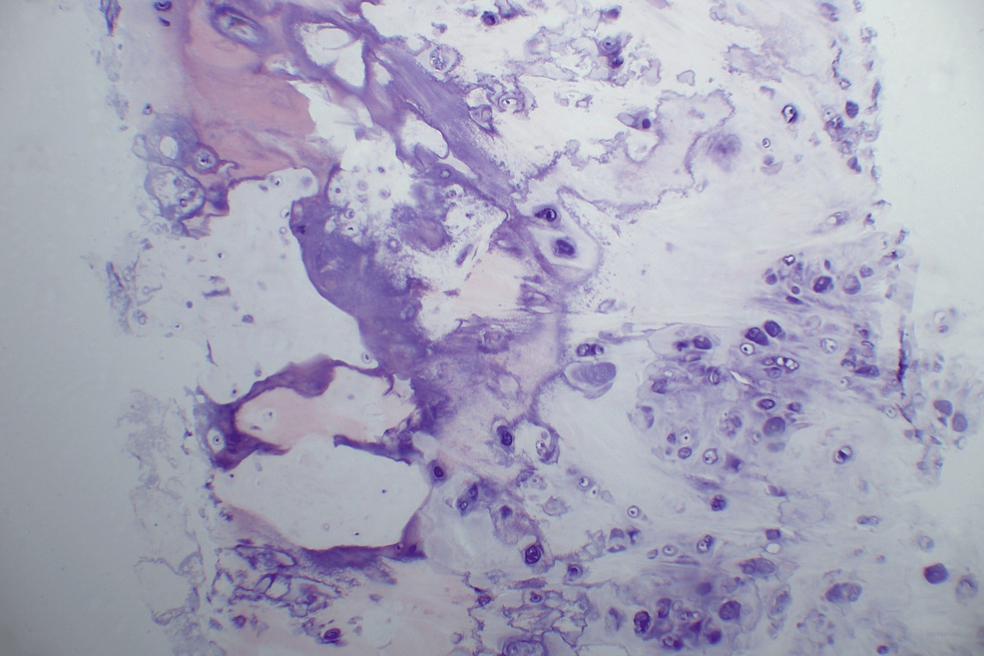
Osteocartlignous proliferation with classic basophilic areas of a “blue bone” formation

**Figure 9:**
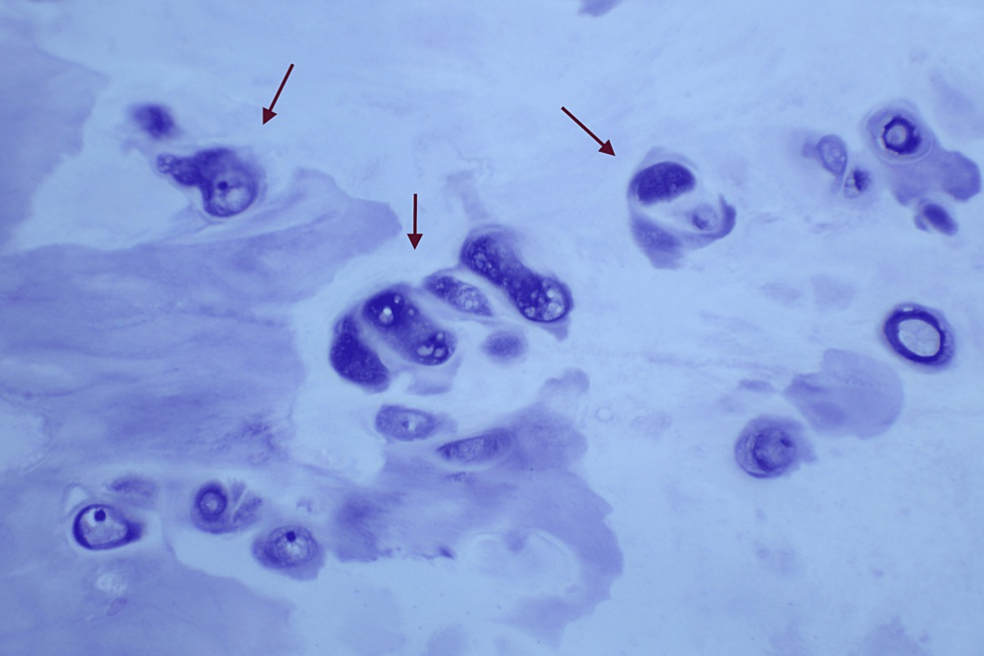
Microscopic examination reveals enlarged chondrocytes with mild to moderate atypia in the form of enlargement and binucleation, but no mitosis was seen

The patient was operated on under local anesthesia with a tourniquet applied on the arm. A lazy S-shaped incision was made just over the swelling using a 15 size blade. Sharp dissection was carried out using a tenotomy scissors. A pearly white swelling was found in the subfascial plane just lying over the periosteum of the fifth metacarpal (Figure 3). The soft tissue attachments were released and the swelling propped out (Figure 4). The swelling was lobulated and contained apparently two types of tissue similar to cortical bone and cartilage (Figure 5). The excision was very easy as the swelling was not firmly adherent to the surrounding tissues. The tourniquet was released and hemostasis achieved. The wound was closed in two layers (Figure 6). A compression dressing was applied, and the patient was discharged to home on the same day. The specimen was sent for histopathological examination, which was later reported to be a Nora’s lesion with the presence of fibrous tissue, bone, and cartilage harboring enlarged binucleated bizarre chondrocytes (Figures 7-9).

## Discussion

BPOP, known as Nora's lesion, is a rare benign growth originating from the osteochondromatous tissue [2]. This condition primarily affects the bones in the distal extremities of young adults, occurring equally in both males and females [3]. It is more prevalent in the hands, particularly in the proximal and middle phalanges, and metacarpal or metatarsal bones, being four times more common in the hands than in the feet [3]. Clinically, Nora's lesions manifest as mildly painful swellings in the hands or feet, gradually increasing in size over several months to years [2]. Typically, there is no history of trauma, although stiffness in nearby joints or other mechanical symptoms might be present [1,5]. In our case, the patient experienced painless swelling. The exact cause of Nora's lesions remains unknown; some theories suggest a reparative process following periosteal trauma, although this was not evident in our patient.

Despite its benign nature and lack of metastasis, Nora's lesions exhibit aggressive local growth patterns [4]. On plain radiography, Nora's lesions appear as well-defined outward growths. They can be distinguished from osteochondromas by their lack of connection with the underlying medulla and absence of cortical flaring [2,5]. Microscopically, Nora's lesions display irregular maturation of hypercellular cartilage into the bone, resulting in a bone with a distinctive blue appearance ("blue bone") [5]. The cartilage lobules contain abnormal binucleate chondrocytes, and benign spindle cell proliferation occurs between the bony trabeculae [7]. Due to its disorganized structure and cytological abnormalities, Nora's lesions can be misidentified as chondrosarcoma or osteosarcoma.

The treatment involves a straightforward excision; however, the recurrence rate is high. Nora and colleagues reported a 51% initial recurrence rate, with 22% experiencing a second recurrence [1]. Most recurrences occur within two years of excision. A wide excision is likely curative in preventing further occurrences.

## Conclusions

The diagnosis of Nora's lesions is challenging as it can be confused with other malignant conditions. When assessing osteogenic or chondromatous growths in the hand, it is crucial to include this rare condition in the differential diagnosis. Surgeons must be aware of the tumor's presence to conduct the necessary resection accurately and routinely advise the histopathological examination to confirm the diagnosis.
